# Immediate Skin-to-Skin Contact at Very Preterm Birth and Neurodevelopment the First Two Years: Secondary Outcomes from a Randomised Clinical Trial

**DOI:** 10.3390/children12080986

**Published:** 2025-07-27

**Authors:** Karoline Lode-Kolz, Wibke Jonas, Hanne Brit Hetland, Karen Helene Hovland Instebø, Henriette Tokvam, Hanne Pike, Siri Lilliesköld, Stina Klemming, Agnes Linnér, Ulrika Ådén, Siren Rettedal

**Affiliations:** 1 Department of Paediatrics, Stavanger University Hospital, 4068 Stavanger, Norway; hanne.markhus.pike@sus.no; 2 Faculty of Health Sciences, University of Stavanger, 4036 Stavanger, Norway; siren.irene.rettedal@sus.no; 3 Department of Clinical Neurophysiology, Stavanger University Hospital, 4068 Stavanger, Norway; 4 Department of Women’s and Children’s Health, Karolinska Institute, 17177 Stockholm, Sweden; wibke.jonas@ki.se (W.J.); siri.lillieskold@ki.se (S.L.); ulrika.aden@ki.se (U.Å.); 5 Department of Biostatistics, Stavanger University Hospital, 4068 Stavanger, Norway; hanne.brit.hetland@sus.no; 6 Department of Psychiatry, Stavanger University Hospital, 4068 Stavanger, Norway; karen.helene.hovland.instebo@sus.no; 7 Department of Physiotherapy, Stavanger University Hospital, 4068 Stavanger, Norway; henriette.tokvam@sus.no; 8 Department of Neonatology, Astrid Lindgren Children’s Hospital, Karolinska University Hospital, 17100 Stockholm, Sweden; agnes.linner@ki.se; 9 Lund-Malmö NIDCAP Training and Research Centre, Department of Neonatology, Skåne University Hospital, 22242 Lund, Sweden; stina.klemming@skane.se; 10 Department of Clinical Science, Intervention and Technology, Karolinska Institute, 14186 Stockholm, Sweden; 11 Department of Biomedical and Clinical Sciences, Linköping University, 58185 Linköping, Sweden; 12 Department of Simulation-Based Learning, Stavanger University Hospital, 4036 Stavanger, Norway

**Keywords:** skin-to-skin contact, kangaroo mother care, prematurity

## Abstract

**Highlights:**

**What are the main findings?**

- In this randomised clinical trial, we found that skin-to-skin contact immediately at birth did not enhance cognition, motor, or social development during the first two years of life but may have been important for language skills.
- Infants having received iSSC showed superior language skills at two years corrected age when adjusted for parents’ education and infants’ sex as compared to controls.

**What is the implication of the main findings?**

- This study along with previous findings from IPISTOSS and other studies on iSSC, support the World Health Organisation guidelines to avoid parental–infant separation by implementing iSSC for low birth weight and preterm infants in all settings. Our findings may indicate a multifactorial effect of iSSC on language acquisition.

**Abstract:**

**Background**: Very preterm infants are at increased risk of impairment. The objective was to explore the effect of immediate parent–infant skin-to-skin contact at very preterm birth on cognition, motor, social, and language development during the two first years. **Methods**: The Immediate Parent-Infant Skin-To-Skin Study (IPISTOSS) was a clinical trial with inclusions between April 2018 to June 2021, in three Scandinavian neonatal intensive care units. Infants were randomised at gestational age 28 + 0 to 32 + 6 weeks plus days, to immediate and continuous skin-to-skin contact at birth or conventional care, during the first six hours of life. **Results**: At three months, 42 infants underwent a General Movement Assessment. At four and 12 months, 69 and 62 infants, respectively, were assessed with the Alberta Motor Infant Scale. At 24 months, language and cognition were tested in 62 infants with the Bayley Scales of Infant and Toddler Development, third edition. Parents completed the Modified Checklist for Autism in Toddlers for 57 infants. There were no significant differences in motor development, cognition, or autism spectrum disorders. A significant difference in language scores in favour of immediate skin-to-skin contact, was found, when adjusted for fathers’ education, mothers’ education, and infants’ sex, Beta (95% CI): 32.00 (7.57, 56.43) *p* = 0.01, 11.51 (8.94, 55.06) *p* = 0.007, and 32.00 (7.85, 56.15) *p* = 0.01, respectively. **Conclusions**: Skin-to-skin contact immediately at birth did not enhance cognition, motor, or social development during the first two years of life but may have been important for language skills. Our findings support the World Health Organisation guidelines recommending iSSC for preterm born infants in all settings.

## 1. Introduction

Approximately 6% of infants in the Nordic countries are born preterm, of whom 0.7–1.3% very preterm at gestational age less than 32 weeks [1]. These infants are at increased risk of developing structural and functional brain abnormalities as the immature nervous system adapts to extra-uterine life [2]. Stressful and painful experiences during the neonatal intensive care unit (NICU) stay may contribute to neurodevelopmental disorders or impairment [3]. Separation from parents at birth and in the NICU deprives the preterm infant of early parental–infant interactions and sensorial stimuli from embodied interactions [4]. Early separation can impact bonding and lead to a dysfunctional parent–infant relationship [5]. Studies of adverse effects of preterm birth show a possible impact on motor development [6], cognition [7], language [8], and autism spectrum disorder (ASD) [9].

Early parent–infant shared attention, shared vocalisations, gazes, and mimics are prerequisites for verbal communication [10]. In the incubator, the infant is less exposed to the mother’s voice, and more to non-meaningful and unpredictable high frequency sounds [11]. Separation of bodies impacts early bidirectional sensorimotor experiences, possibly explaining some of the sensory processing difficulties commonly seen in preterm born infants [12]. An infant who responds little or negatively to auditory stimuli may influence the mothers’ affectionate behaviours, involving touch and sound, and alter a mother–infant bio-behavioural synchrony [13]. Prematurity has been associated with an increased risk of altered maternal sensitivity with less responsiveness and a more intrusive interaction style, as the preterm infant is perceived as less interactive and less responsive [14]. On a longer term, suboptimal language skills can affect social relationships and scholar achievement [8].

Parent–infant skin-to-skin contact (SSC) can be neuroprotective inducing long-lasting favourable effects beyond childhood [15]. SSC is a reciprocal sensory stimulation between the parent and the newborn, triggering activity in the insular cortex, a brain region associated with social bonding, and the somatosensory cortex, critical for cognitive functions [5]. During SSC, oxytocin is released in the parental and infant brain and blood circulation [16], modulating attachment, parenting behaviours, anxiety, and stress [17,18]. A meta-analysis reported beneficial effects of SSC on cognitive and motor development [19]. A cohort study comparing SSC initiated within 72 h after preterm birth versus SSC initiated later reported improved cognition, language, and adaptive behaviour at 12 months of corrected age [20]. A neuroimaging study showed larger volumes of grey matter, cerebellum, and basal ganglia, as well optimised white matter organisation in young adults born preterm having received late SSC [21].

The risk of developing ASD increases with the degree of prematurity [9]. The aetiology could be a complex interaction between genetic and environmental factors, even though many questions remain unanswered. Stressful stimuli could be associated to epigenetic alterations and affect the rapidly developing neuroendocrine and central nervous systems, with increased risk of neurodevelopmental disorders [22]. A secondary analysis on extremely preterm infants and time spent in SSC during the first 30 days of life found higher social competence and lower dysregulation behaviour at two years of age in infants receiving more SSC and shorter latency between SSC sessions [23].

Concerns about clinical instability and risk of intraventricular haemorrhage (IVH) as the head is turned sideways in the SSC prone position may partly explain why few very preterm infants receive SSC during the first day of life [24,25]. A Cochrane review explored supine position–head midline versus other head positions for preventing intraventricular haemorrhage (IVH) in very preterm infants, and found no significant differences [26].

The aim of the present study was to investigate the effect of parent–infant immediate SSC (iSSC) and during the first six hours after very preterm birth, on neurological outcome, measured by motor, cognitive, and language development and ASD screening throughout the first two years of life, as compared to SSC initiated later. Our hypothesis is that iSSC at preterm birth could be favourable for neurodevelopment. The present study reports on secondary outcomes from the Immediate Parent-Infant Skin-To-Skin Study (IPISTOSS) [27].

## 2. Materials and Methods

### 2.1. Study Design, Setting, and Participants

IPISTOSS was a randomised clinical trial (RCT) with two non-blinded parallel arms. Enrolment was conducted between April 2018 and June 2021 at the NICUs in Stavanger University Hospital, Norway, and Huddinge and Danderyd at Karolinska University Hospital, Sweden. Women hospitalised for threatening preterm birth at 28 + 0 to 32 + 6 weeks plus days in pregnancy were screened by staff, and informed written consent was obtained from both parents. Singletons and twins with a second caregiver present could be included, regardless of mode of birth. Higher-order births, infants with congenital infection, or infants with major malformations were excluded. Electronic randomisation before birth was performed (Karolinska Trial Alliance, www.randomize.net) with strata for the three sites and for gestational age (GA) 28 + 0 to 30 + 6 and GA 31 + 0 to 32 + 6 weeks and days. All infants were cared for according to prevailing national and European guidelines [28].

### 2.2. Intervention, Primary Outcome

For infants allocated to the intervention group, iSSC was started as soon as possible after birth. The infant was dried, placed naked on the parents’ bare chest and covered with preheated cloths. Most infants were placed in a prone position with the head to one side. After placement of umbilical catheters, a lateral side lying position was sometimes used. After vaginal birth, iSSC was initiated with the mother. Following maternal conditions, including caesarean, contraindicating iSSC with the mother, iSSC was initiated with the other parent and then continued with the mother as soon as she was available. Transfer to the NICU was carried out in SSC with mother whenever possible or with the other parent, aiming for continuous SSC for the first six hours of life. Placement of monitoring equipment and all medical procedures was performed during SSC, except for endotracheal intubation, placement of umbilical catheters, and radiology examinations. The infant was then temporarily placed in an incubator or under a radiant warmer for the duration of the procedure.

Infants in the conventional care (CC) group were stabilised on a resuscitaire or in an Omnibed incubator (GE Healthcare, Laurel, MD, USA) and transported to the NICU. Parents could be at the bedside, were able to hand hold the infant and were actively included in the care of their infant. Intermittent SSC was initiated after the first six hours after birth following standard protocols.

During the first six hours and thereafter throughout the first eight days, time in SSC was logged with 15 min accuracy [29].

### 2.3. Neurodevelopment Assessment, Secondary Outcomes

The infants came to follow-up at 3 to 4 months corrected age, 12 months corrected age, and 24 months corrected age.

#### 2.3.1. General Movement Assessment (GMA)

At three months corrected age, the Norwegian cohort of infants was assessed according to the GMA. They were video recorded from five to ten minutes to study the quality of spontaneous fidgety movements. Absence of fidgety movements can be predictive of cerebral palsy and atypical movements might be useful for the identification of early signs of neurodevelopmental disorders [30,31]. The videos were made and classified by a paediatric physiotherapist, certified by the General Movement Trust in the Prechtl methodology, with GMA Basic and Advanced courses, and who was blinded to randomisation.

#### 2.3.2. Alberta Motor Infant Scale (AIMS)

Motor development was assessed by paediatricians or physiotherapists at 4 and at 12 months corrected age, in both countries. An AIMS score between 5th to 10th percentile was classified as delayed, and a score at or below the 5th percentile was classified as abnormal [32]. Assessors were blinded to randomisation in the Norwegian cohort. In the Swedish cohort, assessors were partly blinded, as some were clinicians in the NICU.

#### 2.3.3. Bayley Scales of Infant and Toddler Development, Third Edition (BSID-III)

At 24 months corrected age, the toddler’s cognitive and language skills were assessed by two child psychologists, one in Norway and one in Sweden, certified in administering the BSID-III [33]. Composite index scores were calculated for cognition and language (expressive and receptive). Assessors were blinded to randomisation.

#### 2.3.4. Modified Checklist for Autism in Toddlers (M-CHAT)

The questionnaire of 23 items was completed by the parent at 24 months corrected age, in both countries, with scores from 0 to 23, were 0 means no failed questions and 23 means all questions failed. A total score of 0–2 places the child at low risk for ASD, 3–7 at medium risk, and 8–20 at high risk [34].

### 2.4. Data Analysis

Statistical analyses were performed employing IBM SPSS Statistics 26, Stata/SE 18.0, and R 4.3.2. A sample size of 150 patients was determined by the IPISTOSS primary outcome, with cardio-respiratory stabilisation during the first six hours. For this secondary outcome, a subsample of about 50 infants was estimated to be adequate [27,35]. Background characteristics were assessed using Chi-square test for categorical variables and Student’s *t*-test for continuous variables. Background characteristics were analysed independently for the 4-, 12-, and 24-month follow-ups and further analyses non-adjusted for strata, as there were some dropouts that could have potentially influenced the composition of study groups over time. The background characteristics that were significantly different between allocations were adjusted for in data analysis at each time-point for follow-up. Data analyses were carried out according to randomisation group, with intention to treat. All models were adjusted for twins using clustered standard error and the statistical significance level was set to two-sided *p*-value < 0.05. Supplementary analyses for all assessments were thereafter run to see whether an adjustment for accumulated time in SSC after the first 6 h, at 72 h, and at 8 days modified our results.

#### 2.4.1. AIMS

Linear regression analysis was used for normally distributed data.

#### 2.4.2. BSID-III

Linear regression analysis was used for normally distributed data and quantile regression for non-normally distributed data. Separate preliminary Pearson correlations were run to examine associations between fathers’ and mothers’ education and language scores separately for each group.

#### 2.4.3. M-CHAT

A Poisson regression analysis was used to examine the difference between the iSSC and CC group.

## 3. Results

**Figure 1:**
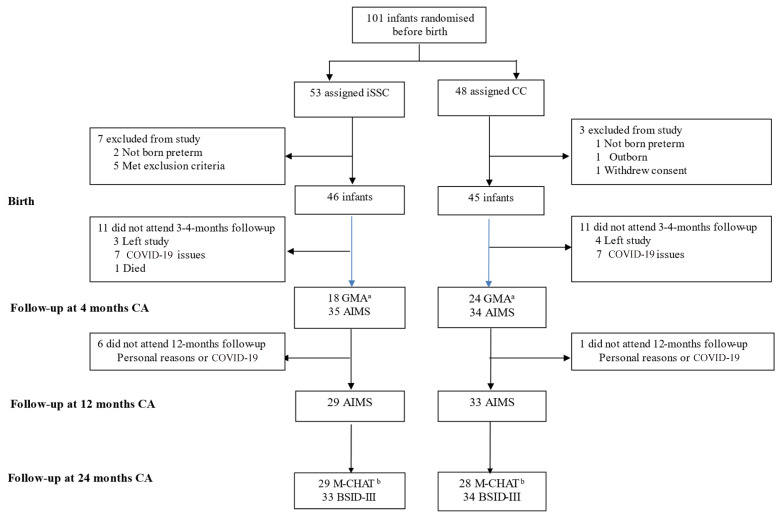
Trial Profile of the 91 infants included, 1 infant from the CC group was diagnosed with IVH grade I at five days of age, with no cases of IVH grade II–IV. Abbreviations: iSSC, immediate Skin-to-Skin Contact; CC, Conventional Care; CA, Corrected Age; GMA, General Movement Assessment; AIMS, Alberta Motor Infant Scale; M-CHAT, Modified Checklist for Autism in Toddlers; BSID-III, Bayley Scales of Infant and Toddler Development, Third edition. ^a^ Norwegian cohort infants, GMA done only in Norway. ^b^ Some infants only attended the BSID-III assessment, but did not show up for medical check and M-CHAT, due to fatigue, illness or personal reasons.

Ninety-one infants were included in the IPISTOSS, 46 randomised to iSSC and 45 to CC (see Figure 1).

Except infant sex, with a significantly larger proportion of boys in the SSC group, *p* = 0.002, baseline characteristics were equally distributed at inclusion (see Table 1(a)). During the first six hours of life, median infant SSC durations with interquartile range (IQR) in the SSC and CC groups were 5.0 (4.4, 5.5) and 0.0 (0.0, 0.0) h, respectively. In the SSC group, median (IQR) maternal SSC duration was 0.6 (0.0, 2.8) h and median (IQR) paternal SSC duration was 3.4 (2.3, 4.8) h. The infants in the iSSC group had a higher accumulated median time of SSC compared to the control group both at 72 h and 8 days after birth (see Table 1(b)).

### 3.1. Follow-Up at Three to Four Months Corrected Age

#### 3.1.1. Background Characteristics

There were significantly more boys and primiparity in the iSSC group as compared to the CC group, namely, 47.1% versus 71.4%, *p* = 0.039 and 74.3% versus 50.0%, *p* = 0.037, respectively. More fathers had a university level education and there were more twins in the CC as compared to iSSC group, namely, 76.5% versus 48.6%, *p* = 0.040 and 50.0% versus 25.70%, *p* = 0.037. Table 2(a).

#### 3.1.2. GMA

In total, 18 infants in the iSSC and 24 infants in the CC groups were assessed by GMA. All 42 infants (100%) demonstrated normal fidgety movements, either continuous (F++) or intermittent (F+), with no abnormal findings.

#### 3.1.3. AIMS

In total, 69 infants were assessed by AIMS at four months, with 35 in the iSSC and 34 in the CC groups. One infant in the CC group scored abnormally, i.e., less than or equal to the fifth percentile, and two infants in the iSSC group scored delayed, i.e., between the sixth to tenth percentile, without significant differences in adjusted means (95% CI) 0.23 (−1.66–2.11) *p* = 0.81 (see Table 3(a)).

### 3.2. Follow-Up at 12 Months Corrected Age

#### 3.2.1. Background Characteristics

There were significantly more primiparity in the iSSC group as compared to the CC group, i.e., 72.4% versus 45.5%, *p* = 0.03 respectively. The infants in the iSSC group were slightly younger in terms of corrected age at the 12-month follow-up than the infants in the CC group, median (95% CI), 12.00 (11.81–12.00) months versus 12.13 (12.00–12.50) months, *p* = 0.007. Table 2(b).

#### 3.2.2. AIMS

In total, 62 infants were assessed with AIMS at 12 months corrected age, with 29 in the iSSC and 33 in the CC groups. Four infants in the CC group and two in the SSC group scored abnormal, less than, or equal to the fifth percentile. One infant in the CC group scored delayed, i.e., between the sixth to tenth percentile, and none in the SSC-group. No significant difference was found in adjusted means (95% CI) 0.84 (−4.04–5.71) *p* = 0.73 (see Table 3(a)).

### 3.3. Follow-Up at 24 Months Corrected Age

#### 3.3.1. Background Characteristics

Background characteristics of infants and parents were equally distributed at BSID-III and M-CHAT assessments, except a larger proportion of fathers having a university education level in the CC group as compared to iSSC group, namely, 82.4% versus 39.4%, *p* < 0.001 and 78.6% versus 44.8% respectively, *p* = 0.020. There was a non-significant difference in the distribution of infants’ sex at 24 months, *p* = 0.17, with more boys in the iSSC group. In addition, at the M-CHAT assessment, a significant higher proportion of primiparity was found in the iSSC as compared to CC groups, namely, 74.1% versus 45.8%, *p* = 0.039. Table 2(c).

#### 3.3.2. BSID-III

In total, 62 infants were assessed by BSID-III, with 31 in the iSSC and 31 in the CC groups. Two of these infants completed the cognitive assessment, but failed to participate in the language assessment, both from the CC group. Five additional infants were present at the 24-month follow-up, but could not be assessed due to behavioural issues, with two from the iSSC and three from the CC groups.

For BSID-III language scores, there were no difference in adjusted medians (95% CI) 24.00 (3.09–51.09), *p* = 0.08. However, a statistically significant difference in language scores in favour of iSSC was found when adjusted for fathers’ or mothers’ education, with differences in adjusted medians (95% CI) 32.00 (7.57, 56.43), *p* = 0.01 and 11.51 (8.94, 55.06), *p* = 0.007, respectively. This persisted when adjusted for total accumulated SSC time, with differences in adjusted medians (95% CI), i.e., at 72 h, 30.21 (6.25, 54.17) *p* = 0.014, and 8 days, 31.12 (9.47, 52.78) *p* = 0.006, for fathers’ education and at 72 h for mothers’ education, 29.32 (9.00, 49.65) *p* = 0.006. A significant difference also emerged in favour of iSSC when adjusted for infants’ sex, 32.00 (7.85, 56.15) *p* = 0.01. No significant differences between study groups were found in BSID-III cognitive scale (see Table 3(b)).

#### 3.3.3. M-CHAT

The parents of 57 infants completed the M-CHAT questionnaire, with 29 in the iSSC and 28 in the CC groups, with scores showing a median (95% CI, range) of 0.00 (0.00–2.75, 0.00–12.00) and 0.00 (0.00–1.00, 0.00–11.00), respectively. Seven children had scores placing them at medium risk for ASD, with six in the iSSC group and one in the CC group. Two children had scores placing them at high risk, with one from the iSSC group and one from the CC group. No significant differences emerged between groups, ratio of means (95%) 1.81 (0.56–5.86), *p* = 0.325 (Table 4).

## 4. Discussion

In this study, very preterm infants receiving SSC during the first six hours after birth showed no significant differences in language or motor skills, cognition, or ASD during the two years follow-up as compared to controls. When adjusted for parents’ education and infants’ sex the iSSC group showed superior language skills at two years corrected age. Our findings suggest that SSC immediately at birth and during the first hours is safe regarding neurological outcome and might be important for language development.

A possible mechanism of favourable language acquisition is early parental–infant interaction and bonding as well as early exposure to parental language, enhancing non-verbal and verbal communication. This hypothesis is supported by a previous publication from the IPISTOSS group, reporting that iSSC significantly enhanced the quality of mother–infant interactions at four months corrected age, with increased infant communicative and social skills [36]. Similar findings have been reported with SSC starting 45 min after birth for a duration of 60 min, at gestational age 25 to 32 weeks [37].

Already in the intrauterine environment, the developing foetus can perceive attenuated low-frequency sounds, important for the development of auditory pathways [38]. During immediate and continuous SSC, the auditory pathways will process age-appropriate stimuli. Systematic exposure to maternal voice and heartbeat for newborn preterm infants as compared to conventional care has shown an effect on auditory cortex plasticity, demonstrating a significantly larger auditory cortex bilaterally [39]. Our findings align with previous studies demonstrating that exposing preterm infants to parents’ voices in the NICU improves BSID-III language scores [40].

Becoming a parent is accompanied by physiological and endocrinological transformations [41]. Plasticity and long-lasting modifications in the mother’s somatosensory and auditory cortex contribute to increased responsiveness to infant cues [42]. Mother’s sensitivity or her ability to recognise her infant’s behaviour and adapt moment-by-moment to her infant’s needs is an important component of the bio-behavioural synchrony [43]. Alterations in mothers’ sensitivity due to depression at preterm birth might affect caregiving, interaction, and bonding [44]. A previous randomised controlled trial (RCT) from Germany showed that delivery room SSC in very preterm born infants reduces the risk of early post-partum maternal depression and impaired bonding at 6 months, promoting mother–infant interaction [37], important for non-verbal and verbal communication. In our trial, iSSC significantly reduced symptoms of depression in mothers and anxiety in fathers at one week after birth, as well as symptoms of depression and anxiety in fathers at term-corrected age [45]. As iSSC was provided mostly by the fathers, it is possible that father–infant iSSC may have enhanced early father–infant bonding, impacting language development.

For mother–child dyads, it has been shown that maternal level of education relates to children’s language skills, also after preterm birth [46]. There is growing evidence for paternal educational level influencing language development [47,48], as supported by our findings. In our trial, the fathers from the CC group had a higher level of education, possibly impacting the infants’ language development, with no significant differences between study groups. After adjustment for paternal education level, the iSSC group showed superior language scores. Even though there were no significant differences in level of maternal education between study groups, we did an explorative analysis adjusting for mothers’ education and found significant differences in favour of iSSC.

Infants’ sex might impact early language acquisition. Cohort studies on both preterm and term born infants assessed at two years of age have shown language scores in favour of girls [49,50]. In our trial, at 24 months, there was a non-significant higher number of boys in the iSSC group as compared to the CC group. This may have influenced our results. Explorative analysis adjusting for infants’ sex, demonstrated significant differences in favour of iSSC, with higher language scores in this group.

The absolute risks of ASD in infants born within GA week 22–31 and GA week 32–36 in Scandinavia is estimated to be 1.67% and 1.08% [9]. In our trial, the infants were only followed up to the corrected age of two years. ASD is often diagnosed later, during childhood or adulthood [9], and we might not have identified all the infants at risk. A higher rate of ASD in males in the general population might explain the non-significant differences between our study groups, as there were more boys in the iSSC group [51]. However, our study lacks statistical power to conduct subanalyses on the association between GA, ASD, and sex.

No IVH was observed in the iSSC group, despite prone body position with head turned to the side during the iSSC intervention, in line with previous publications [25,26,52].

A RCT reporting on 2 h of maternal–infant SSC in the labour ward at preterm birth did not find a significant effect on neurodevelopmental outcomes at two to three years of age. However, the results were adjusted only for maternal education, with no available data on paternal education or additional socioeconomic variables [53].

Strengths of the study were the study design with randomisation and strata for age and sites, reducing the risk of selection bias. There were only minor variations in characteristics between children analysed and drop-outs at two years corrected age. All participants, independently of study group, received the same medical care; only the place of care differed, following a robust study protocol. The intervention was implemented to a high degree, with a median (IQR) infant SSC duration of 5.0 (4.5, 5.5) h.

However, there were limitations. The simple size was smaller than initially projected, due to a preliminary analysis conducted in June 2021 following COVID-19 pandemic restrictions in recruitment and follow-up. Recruitment to IPISTOSS was terminated October 2021 in agreement with the data safety monitoring board, as iSSC showed a significant beneficial effect on the primary outcome of infant cardiorespiratory stability during the first six hours after birth. During the same period, the WHO published results from their iSSC trial demonstrating significant reduction of mortality and morbidity at very preterm birth. The RCT was terminated prematurely due to the significant benefice of the intervention [54]. The COVID-19 pandemic resulted in a high degree of attrition at 4- and 12-month follow-ups due to restriction policies, with families being lost to follow-ups when this was possible again at 12 and 24 months.

## 5. Conclusions

In this RCT, immediate and continuous SSC for 6 h at very preterm birth did not show significant differences regarding motor, cognitive, language, and ASD during the first two years as compared to control infants. When adjusted for parents’ education and infants’ sex, iSSC infants showed superior language skills at two years corrected age. Our findings may indicate a multifactorial effect of iSSC on language acquisition. This study, along with previous findings from IPISTOSS and other studies on iSSC, support the WHO guidelines for care of the preterm or low-birth-weight infant [55,56] and avoid parental–infant separation by implementing iSSC in all settings.

## Figures and Tables

**Table 1:** Study cohort at birth.

	iSSCN = 46	CCN = 45
** (a). Background characteristic **		
Gestational Age, days, median (IQR)	220.50 (2013.25–226.00)	222.00 (207.00–226.00)
Birth Weight, grams, mean (range)	1572 (702–2352)	1495 (555–2440)
Sex, female, n (%)	13 (28)	27 (60)
Apgar score, 1 min, median (mean)	8 (7.4)	8 (7.4)
Apgar score, 5 min, median (mean)	9 (8.2)	9 (8.6)
Apgar score, 10 min, median (mean)	10 (9.3)	9 (9.0)
Singleton, n (%)	30 (65)	26 (58)
Antenatal corticosteroids, n (%)	46 (100)	43 (96)
Primiparous, n (%)	27 (71)	16 (46)
Maternal age, mean (SD, range)	31 (5, 21–40)	32 (5, 22–45)
** (b). Time spent in skin-to-skin contact **	** median (IQR) **	** median (IQR) **
Total hours mother–infant SSC h0–h6, n = 91	0.63 (0.00–2.81)	0.00 (0.00–0.00)
Total hours father–infant SSC h0–h6, n = 91	3.38 (2.25–4.78)	0.00 (0.00–0.00)
Accumulated hours in SSC for infants h0–h6, n = 91	5.00 (4.44–5.50)	0.00 (0.00–0.00)
Total hours mother–infant SSC h7–h72, n = 89	9.00 (6.33–14.63)	5.75 (3.63–9.75)
Total hours father–infant SSC h7–h72, n = 89	8.63 (2.75–11.99)	3.0 (0.00–4.87)
Accumulated hours in SSC for infants h7–h72, n = 89	17.13 (10.56–25.44)	10.55 (5.75–13.75)
Total hours mother–infant SSC h7–day7, n = 85	28.50 (20.75–41.25)	19.58 (16.04–28.38)
Total hours father–infant SSC h7–day7, n = 85	23.85 (11.56–31.45)	12.25 (6.04–18.13)
Accumulated hours in SSC for infants h7–day7, n = 85	53.13 (37.75–70.29)	36.45 (25.98–43.24)

Abbreviations: iSSC, Immediate Skin-to-Skin Contact; CC, Conventional Care; N, total number of infants; n, number of infants; IQR, interquartile range; SD, standard deviation; SSC, Skin-to-Skin Contact; h, hour.

**Table 2:** Background characteristics at 4-, 12-, and 24-month follow-up.

	(a). 4 Months AIMS		(b). 12 Months AIMS		(c). 24 Months BSID-III/ M-CHAT ^a^	
	iSSC N = 35	CC N = 34	iSSC N = 29	CC N = 33	iSSC N = 31/28 ^a^	CC N = 31/29 ^a^
GA at birth, days, median (IQR)	222.00 (214.00–226.00)	220.00 (205.00–224.25)	216.00 (212.00–223.50)	218.00 (205.00–222.50)	220.00 (212.50–225.50)	220.00 (205.00–225.25)
CA, months, at follow-up, median (IQR, range)	4.00 (3.25–4.00, 2.75–4.50)	4.00 (3.50–4.50, 2.75–4.75)	12.00 (11.81–12.00, 11.50–13.00)	12.13 (12.00–12.50, 11.75–14.25)	24.00 (24.00–25.00, 23.00–28.00)	24.00 (24.00–25.25, 23.00–28.00)
Sex, boy, n (%)	25 (71)	16 (47)	21 (72)	16 (49)	21 (64)	16 (47)
Twin, n (%)	9 (25)	17 (50)	9 (31)	15 (45)	11 (33)	18 (53)
Caesarean, n (%)	22 (62)	26 (76)	20 (69)	26 (79)	21 (63)	26 (76)
Birthweight, grams, mean (SD)	1560.89 (399.90)	1458.56 (417.15)	1486.79 (339.98)	1420.55 (382.51)	1517.52 (393.33)	1490.14 (349.85)
Apgar 5 min, median (IQR)	9.00 (8.00–10.00)	9.00 (8.00–9.25)	9.00 (8.00–9.00)	9.00 (8.00–9.00)	9.00 (8.00, 10.00)	9.00 (8.50–10.00)
Antenatal corticosteroid, n (%)	35 (100)	34 (100)	29 (100)	33 (100)	33 (100)	34 (100)
Preeclampsia, n (%)	10 (29)	12 (35)	8 (28)	14 (42)	9 (27)	11 (32)
Primiparity, n (%)	26 (74)	17 (50)	21 (72)	15 (46)	24 (73)/ 20 (74) ^a^	17 (50)/ 11 (46) ^a^
Maternal age, years, mean (SD)	31.46 (4.39)	32.21 (5.17)	31.28 (3.66)	31.58 (3.86)	31.34 (3.79)	32.6 (4.87)
Paternal age, years, mean (SD)	32.80 (4.49)	34.68 (4.97)	32.80 (4.49)	34.68 (4.97)	32.91 (3.78)	34.06 (5.07)
Cohabiting parents, n (%)	32 (94)	31 (93)	29 (100)	33 (100)	32 (97)	32 (94)
University education of mother, n (%)	22 (69)	26 (76)	20 (69)	25 (75)	20 (61)	27 (79)
University education of father, n (%)	17 (49)	26 (77)	15 (51)	24 (72)	13 (39)/ 13 (45) ^a^	28 (82)/ 22 (79) ^a^
Mental health diagnosis mother, n (%)	5 (15)	2 (5)	2 (7)	2 (6)	5 (15)	3 (9)
Mother tongue other ^b^, n (%)	3 (9)	5 (15)	4 (14)	5 (15)	5 (15)	7 (21)
Father tongue other ^c^, n (%)	4 (11)	7 (21)	5 (17)	5 (15)	3 (9)	5 (15)

Abbreviations: AIMS, Alberta Infant Motor Scale; BSID-III, The Bayley Scales of Infant Development III; M-CHAT, Modified Checklist for Autism in Toddlers; iSSC, Immediate Skin-to-Skin Contact; CC, Conventional Care; N, total number infants; n, number infants; GA, Gestational Age; CA, Corrected Age; IQR, interquartile range; SD, standard deviation; CI, confidence interval; *p*, *p*-value. ^a^ M-CHAT background values, mentioned when significant, ^b^ Mothers’ native language, other than Norwegian or Swedish, ^c^ Fathers’ native language, other than Norwegian or Swedish.

**Table 3:** AIMS and BSID-III: Difference in scores between groups regarding the iSSC group, mediated by accumulated time in SSC at 72 h ^a^ and 8 days ^b^.

(a). AIMS	4 Months N = 69 (iSSC = 35, CC = 34)			12 Months N = 62 (SSC = 29, CC = 33)		
	Beta	95 CI	*p*	Beta	95% CI	*p*
Randomisation to iSSC	0.23	−1.66–2.11	0.81	0.84	−4.04–5.71	0.73
72 h	−0.07	−2.04–1.89	0.94	1.46	−3.90–6.81	0.59
8 days	0.06	−2.08–2.20	0.96	0.61	−5.05–6.27	0.83
adjusted for sex	0.04	−1.84–1.92	0.96	1.07	−4.10–6.24	0.68
72 h	−0.21	−2.22–1.81	0.84	1.58	−3.95–7.12	0.57
8 days	−0.19	−0.69–3.17	0.86	0.79	−5.10–6.68	0.79
adjusted for education father	0.07	−1.95–2.10	0.95	0.52	−4.57–5.61	0.84
72 h	−0.17	−2.20–1.86	0.87	0.94	−4.54–6.42	0.73
8 days	0.06	−2.11–2.23	0.95	0.15	−5.55–5.84	0.96
adjusted for education mother	0.29	−1.60–2.18	0.79	0.90	−4.07–5.87	0.72
72 h	−0.06	−2.06–1.94	0.95	1.54	−3.90–6.99	0.57
8 days	0.01	−2.11–2.14	0.99	0.58	−5.10–6.26	0.84
adjusted for primiparity	0.83	−0.92–2.58	0.35	0.93	−3.72–5.57	0.69
72 h	−2.32	−1.39–2.51	0.57	1.63	−3.18–6.43	0.50
8 days	0.66	−1.41–2.74	0.53	0.85	−4.44–6.14	0.75
adjusted for corrected age	NA	NA	NA	5.20	−0.85–11.24	0.09
72 h	NA	NA	NA	5.70	−0.94–12.34	0.09
8 days	NA	NA	NA	5.42	−1.28–12.12	0.11
**(b). BSID-III**	**Language 24 months** **N = 60 (iSSC = 31, CC = 29)**			**Cognition 24 months** **N = 62 (iSSC = 31, CC = 31)**		
	Beta	95% CI	*p*	Beta	95% CI	*p*
Randomisation to iSSC	24.00	−3.09–51.09	0.08	−2.32	−14.18–9.54	0.70
72 h	22.22	−7.60–52.04	0.14	−9.23	−23.01–4.54	0.19
8 days	24.00	−6.91–54.91	0.13	−8.25	−21.87–5.36	0.23
adjusted for father’s education	32.00	7.57–56.43	0.01	−2.05	−15.40–11.31	0.76
72 h	30.21	6.25–54.17	0.01	−9.31	−24.51–5.89	0.23
8 days	31.12	9.47–52.78	0.01	−6.64	−20.61–7.33	0.35
adjusted for mother’s education	11.51	8.94–55.06	0.01	0.05	−11.68–11.78	0.99
72 h	29.32	9.00–49.65	0.01	−6.97	−19.67–5.73	0.28
8 days	23.89	−0.71–48.49	0.06	−5.90	−18.38–6.58	0.35
adjusted for education father and mother	30.82	8.95–52.69	0.01	NA	NA	NA
adjusted for infants’ sex	32.00	7.85–56.15	0.01	−2.46	−14.00–9.09	0.67
72 h	30.07	4.77–55.37	0.02	−9.23	−23.12–4.66	0.19
8 days	31.50	6.21–56.79	0.02	−8.21	−21.86–5.43	0.23

Abbreviations: AIMS, Alberta Infant Motor Scale; BSID­III, The Bayley Scales of Infant Development III; *p*, *p*-value; iSSC, Immediate Skin-to-Skin Contact; SSC, Skin-to-Skin Contact; CC, Conventional Care; N, total number infants; CI, confidence interval; NA, not applicable or not available, ^a^ total accumulated time in SSC between 6 h and 72 h of life, ^b^ total accumulated time in SSC between 6 h and 8 days of life.

**Table 4:** M-CHAT 24 months: Differences in scores between groups.

N = 57 (iSSC = 29, CC = 28)			
	RM	95% CI	*p*
Randomisation to iSSC	1.81	0.56–5.86	0.325
adjusted for infants’ sex	1.66	0.53–5.20	0.388
adjusted for father’s education	1.65	0.57–4.79	0.360
adjusted for mother’s education	1.73	0.57–5.23	0.334
adjusted for primiparity	1.07	0.23–7.22	0.929

Abbreviations: M-CHAT, Modified Checklist for Autism in Toddlers; iSSC, Immediate Skin-to-Skin Contact; SSC, Skin-to-Skin Contact; CC, Conventional Care; N, total number infants; RM, ratio of means; CI, confidence interval; *p*, *p*-value.

## Data Availability

When obtaining consent from the parents included in the study, we have not specifically asked for their permission to share data to the larger scientific community, and therefore, data will not be available.

## Abbreviations

The following abbreviations are used in this manuscript:

IPISTOSS	Immediate Parent-Infant Skin-To-Skin Study
NICU	neonatal intensive care unit
ASD	autism spectrum disorders
SSC	skin-to-skin contact
IVH	intraventricular haemorrhage
iSSC	immediate skin-to-skin contact
RCT	randomised clinical trial
GA	gestational age
CC	conventional care
GMA	General Movement Assessment
AIMS	Alberta Motor Infant Scale
BSID-III	Bayley Scales of Infant and Toddler Development, Third edition
M-CHAT	Modified Checklist for Autism in Toddlers
IQR	interquartile range
WHO	World Health Organisation

## References

[1] Norman M., Padkaer Petersen J., Stensvold H.J., Thorkelsson T., Helenius K., Brix Andersson C., Ørum Cueto H., Domellöf M., Gissler M., Heino A. (2023). Preterm birth in the Nordic countries-Capacity, management and outcome in neonatal care. Acta Paediatr..

[2] Volpe J.J. (2019). Dysmaturation of Premature Brain: Importance, Cellular Mechanisms, and Potential Interventions. Pediatr. Neurol..

[3] Rand K., Lahav A. (2014). Impact of the NICU environment on language deprivation in preterm infants. Acta Paediatr..

[4] La Rosa V.L., Geraci A., Iacono A., Commodari E. (2024). Affective Touch in Preterm Infant Development: Neurobiological Mechanisms and Implications for Child–Caregiver Attachment and Neonatal Care. Children.

[5] Carozza S., Leong V. (2020). The Role of Affectionate Caregiver Touch in Early Neurodevelopment and Parent-Infant Interactional Synchrony. Front. Neurosci..

[6] Vollmer B., Stålnacke J. (2019). Young Adult Motor, Sensory, and Cognitive Outcomes and Longitudinal Development after Very and Extremely Preterm Birth. Neuropediatrics.

[7] Twilhaar E.S., Wade R.M., de Kieviet J.F., van Goudoever J.B., van Elburg R.M., Oosterlaan J. (2018). Cognitive Outcomes of Children Born Extremely or Very Preterm Since the 1990s and Associated Risk Factors: A Meta-analysis and Meta-regression. JAMA Pediatr..

[8] Vandormael C., Schoenhals L., Hüppi P.S., Filippa M., Borradori Tolsa C. (2019). Language in Preterm Born Children: Atypical Development and Effects of Early Interventions on Neuroplasticity. Neural Plast..

[9] Persson M., Opdahl S., Risnes K., Gross R., Kajantie E., Reichenberg A., Gissler M., Sandin S. (2020). Gestational age and the risk of autism spectrum disorder in Sweden, Finland, and Norway: A cohort study. PLoS Med..

[10] McMahon E., Wintermark P., Lahav A. (2012). Auditory brain development in premature infants: The importance of early experience. Ann. N. Y. Acad. Sci..

[11] Sibrecht G., Wróblewska-Seniuk K., Bruschettini M. (2024). Noise or sound management in the neonatal intensive care unit for preterm or very low birth weight infants. Cochrane Database Syst. Rev..

[12] Woolard A., Coleman A., Johnson T., Wakely K., Campbell L.E., Mallise C.A., Whalen O.M., Murphy V.E., Karayanidis F., Lane A.E. (2022). Parent-infant interaction quality is related to preterm status and sensory processing. Infant. Behav. Dev..

[13] Shai D., Belsky J. (2017). Parental embodied mentalizing: How the nonverbal dance between parents and infants predicts children’s socio-emotional functioning. Attach. Hum. Dev..

[14] Hartzell G., Shaw R.J., Givrad S. (2023). Preterm infant mental health in the neonatal intensive care unit: A review of research on NICU parent-infant interactions and maternal sensitivity. Infant. Ment. Health J..

[15] Kostandy R.R., Ludington-Hoe S.M. (2019). The evolution of the science of kangaroo (mother) care (skin-to-skin contact). Birth Defects Res..

[16] Jones C., Barrera I., Brothers S., Ring R., Wahlestedt C. (2017). Oxytocin and social functioning. Dialogues Clin. Neurosci..

[17] Moberg K.U., Handlin L., Petersson M. (2020). Neuroendocrine mechanisms involved in the physiological effects caused by skin-to-skin contact—With a particular focus on the oxytocinergic system. Infant. Behav. Dev..

[18] Uvnäs-Moberg K. (2014). Oxytocin: The Biological Guide to Motherhood.

[19] Akbari E., Binnoon-Erez N., Rodrigues M., Ricci A., Schneider J., Madigan S., Jenkins J. (2018). Kangaroo mother care and infant biopsychosocial outcomes in the first year: A meta-analysis. Early Hum. Dev..

[20] Bisanalli S., Balachander B., Shashidhar A., Raman V., Josit P., Rao S.P. (2023). The beneficial effect of early and prolonged kangaroo mother care on long-term neuro-developmental outcomes in low birth neonates—A cohort study. Acta Paediatr..

[21] Charpak N., Tessier R., Ruiz J.G., Uriza F., Hernandez J.T., Cortes D., Montealegre-Pomar A. (2022). Kangaroo mother care had a protective effect on the volume of brain structures in young adults born preterm. Acta Paediatr..

[22] Casavant S.G., Cong X., Moore J., Starkweather A. (2019). Associations between preterm infant stress, epigenetic alteration, telomere length and neurodevelopmental outcomes: A systematic review. Early Hum. Dev..

[23] Gonya J., Feldman K., Brown K., Stein M., Keim S., Boone K., Rumpf W., Ray W., Chawla N., Butter E. (2018). Human interaction in the NICU and its association with outcomes on the Brief Infant-Toddler Social and Emotional Assessment (BITSEA). Early Hum. Dev..

[24] Linnér A., Lilliesköld S., Jonas W., Skiöld B. (2022). Initiation and duration of skin-to-skin contact for extremely and very preterm infants: A register study. Acta Paediatr..

[25] Johansson M.W., Lilliesköld S., Jonas W., Thernström Blomqvist Y., Skiöld B., Linnér A. (2024). Early skin-to-skin contact and the risk of intraventricular haemorrhage and sepsis in preterm infants. Acta Paediatr..

[26] Romantsik O., Calevo M.G., Bruschettini M. (2020). Head midline position for preventing the occurrence or extension of germinal matrix-intraventricular haemorrhage in preterm infants. Cochrane Database Syst. Rev..

[27] Linnér A., Westrup B., Lode-Kolz K., Klemming S., Lillieskold S., Markhus Pike H., Morgan B., Bergman N.J., Rettedal S., Jonas W. (2020). Immediate parent-infant skin-to-skin study (IPISTOSS): Study protocol of a randomised controlled trial on very preterm infants cared for in skin-to-skin contact immediately after birth and potential physiological, epigenetic, psychological and neurodevelopmental consequences. BMJ Open.

[28] Sweet D.G., Carnielli V., Greisen G., Hallman M., Ozek E., Te Pas A., Plavka R., Roehr C.C., Saugstad O.D., Simeoni U. (2019). European consensus guidelines on the management of respiratory distress syndrome—2019 Update. Neonatology.

[29] Axelin A., Raiskila S., Lehtonen L. (2020). The Development of Data Collection Tools to Measure Parent-Infant Closeness and Family-Centered Care in NICUs. Worldviews Evid. Based Nurs..

[30] Hadders-Algra M. (2021). Early Diagnostics and Early Intervention in Neurodevelopmental Disorders-Age-Dependent Challenges and Opportunities. J. Clin. Med..

[31] Robinson H., Hart D., Vollmer B. (2021). Predictive validity of a qualitative and quantitative Prechtl’s General Movements Assessment at term age: Comparison between preterm infants and term infants with HIE. Early Hum Dev..

[32] Fuentefria R.D.N., Silveira R.C., Procianoy R.S. (2017). Motor development of preterm infants assessed by the Alberta Infant Motor Scale: Systematic review article. J. Pediatr. (Rio J.).

[33] Del Rosario C., Slevin M., Molloy E.J., Quigley J., Nixon E. (2021). How to use the Bayley Scales of Infant and Toddler Development. Arch. Dis. Child. Educ. Pract. Ed..

[34] Gray P.H., Edwards D.M., O’Callaghan M.J., Gibbons K. (2015). Screening for autism spectrum disorder in very preterm infants during early childhood. Early Hum. Dev..

[35] Chi Luong K., Long Nguyen T., Huynh Thi D.H., Carrara H.P., Bergman N.J. (2016). Newly born low birthweight infants stabilise better in skin-to-skin contact than when separated from their mothers: A randomised controlled trial. Acta Paediatr..

[36] Lilliesköld S., Lode-Kolz K., Rettedal S., Lindstedt J., Linnér A., Markhus Pike H., Ahlqvist-Björkroth S., Ådén U., Jonas W. (2023). Skin-to-Skin Contact at Birth for Very Preterm Infants and Mother-Infant Interaction Quality at 4 Months: A Secondary Analysis of the IPISTOSS Randomized Clinical Trial. JAMA Netw. Open.

[37] Mehler K., Hucklenbruch-Rother E., Trautmann-Villalba P., Becker I., Roth B., Kribs A. (2020). Delivery room skin-to-skin contact for preterm infants-A randomized clinical trial. Acta Paediatr..

[38] Pineda R., Kellner P., Guth R., Gronemeyer A., Smith J. (2023). NICU sensory experiences associated with positive outcomes: An integrative review of evidence from 2015–2020. J. Perinatol..

[39] Webb A.R., Heller H.T., Benson C.B., Lahav A. (2015). Mother’s voice and heartbeat sounds elicit auditory plasticity in the human brain before full gestation. Proc. Natl. Acad. Sci. USA..

[40] Caskey M., Stephens B., Tucker R., Vohr B. (2014). Adult talk in the NICU with preterm infants and developmental outcomes. Pediatrics.

[41] Bridges R.S. (2016). Long-term alterations in neural and endocrine processes induced by motherhood in mammals. Horm. Behav..

[42] Kim P., Strathearn L., Swain J.E. (2016). The maternal brain and its plasticity in humans. Horm. Behav..

[43] Kohl J., Dulac C. (2018). Neural control of parental behaviors. Curr. Opin. Neurobiol..

[44] Kim S., Soeken T.A., Cromer S.J., Martinez S.R., Hardy L.R., Strathearn L. (2014). Oxytocin and postpartum depression: Delivering on what’s known and what’s not. Brain Res..

[45] Lilliesköld S., Lode-Kolz K., Westrup B., Bergman N., Sorjonen K., Ådén U., Mörelius E., Rettedal S., Jonas W. (2025). Skin-to-skin contact at birth for very preterm infants and symptoms of depression and anxiety in parents during the first year—A secondary outcome of a randomized clinical trial. J. Affect. Disord..

[46] Asztalos E.V., Church P.T., Riley P., Fajardo C., Shah P.S. (2017). Association between Primary Caregiver Education and Cognitive and Language Development of Preterm Neonates. Am. J. Perinatol..

[47] Teufl L., Deichmann F., Supper B., Ahnert L. (2020). How fathers’ attachment security and education contribute to early child language skills above and beyond mothers: Parent-child conversation under scrutiny. Attach. Hum. Dev..

[48] Vanderauwera J., van Setten E.R.H., Maurits N.M., Maassen B.A.M. (2019). The interplay of socio-economic status represented by paternal educational level, white matter structure and reading. PLoS ONE.

[49] Gayraud F., Lanoë J.L., De Agostini M. (2025). Factors influencing language performance in boys and girls at age 2 in the French ELFE birth cohort. Brain Res..

[50] Peyre H., Hoertel N., Bernard J.Y., Rouffignac C., Forhan A., Taine M., Heude B., Ramus F. (2019). Sex differences in psychomotor development during the preschool period: A longitudinal study of the effects of environmental factors and of emotional, behavioral, and social functioning. J. Exp. Child. Psychol..

[51] Hintz S.R., Kendrick D.E., Vohr B.R., Kenneth Poole W., Higgins R.D. (2006). Gender differences in neurodevelopmental outcomes among extremely preterm, extremely-low-birthweight infants. Acta Paediatr..

[52] Minot K.L., Kramer K.P., Butler C., Foster M., Gregory C., Haynes K., Lagon C., Mason A., Wynn S., Rogers E.E. (2021). Increasing Early Skin-to-Skin in Extremely Low Birth Weight Infants. Neonatal Netw..

[53] Kristoffersen L., Støen R., Bergseng H., Flottorp S.T., Magerøy G., Grunewaldt K.H., Aker K. (2025). Immediate Skin-to-Skin Contact in Very Preterm Neonates and Early Childhood Neurodevelopment: A Randomized Clinical Trial. JAMA Netw. Open.

[54] Arya S., Naburi H., Kawaza K., Newton S., Anyabolu C.H., Bergman N., Rao S., Mittal P., Assenga E., Gadama L. (2021). Immediate “Kangaroo Mother Care” and Survival of Infants with Low Birth Weight. N. Engl. J. Med..

[55] World Health Organization (2022). WHO Recommendations for Care of the Preterm or Low-Birth-Weight Infant.

[56] World Health Organization (2022). Early Essential Newborn Care: Clinical Practice Pocket Guide.

